# Supplementary material for: Age-specific SARS-CoV-2 infection fatality rates derived from serological data vary with income and income inequality
Source: PLoS One. 2023 May 17;18(5):e0285612. doi: 10.1371/journal.pone.0285612 (PMC10191265; doi:10.1371/journal.pone.0285612)

**S1 Figure. Age-specific infection fatality ratio (IFR) of COVID-19 (mean ± 95% CI) on an untransformed scale.** Points are plotted at the midpoint of the age class on the x-axis, and slightly jittered along the x-axis facilitate presentation. Lines show age-specific IFRs for different populations, using confirmed-only COVID-19 deaths where possible. This study is represented in red, as "New York City". Values of 0 are plotted at 0.0001% and labeled 0 to facilitate presentation on a log scale. The upper bound on the 90+ class for Sweden extends to 29.5% but is represented here as 20%

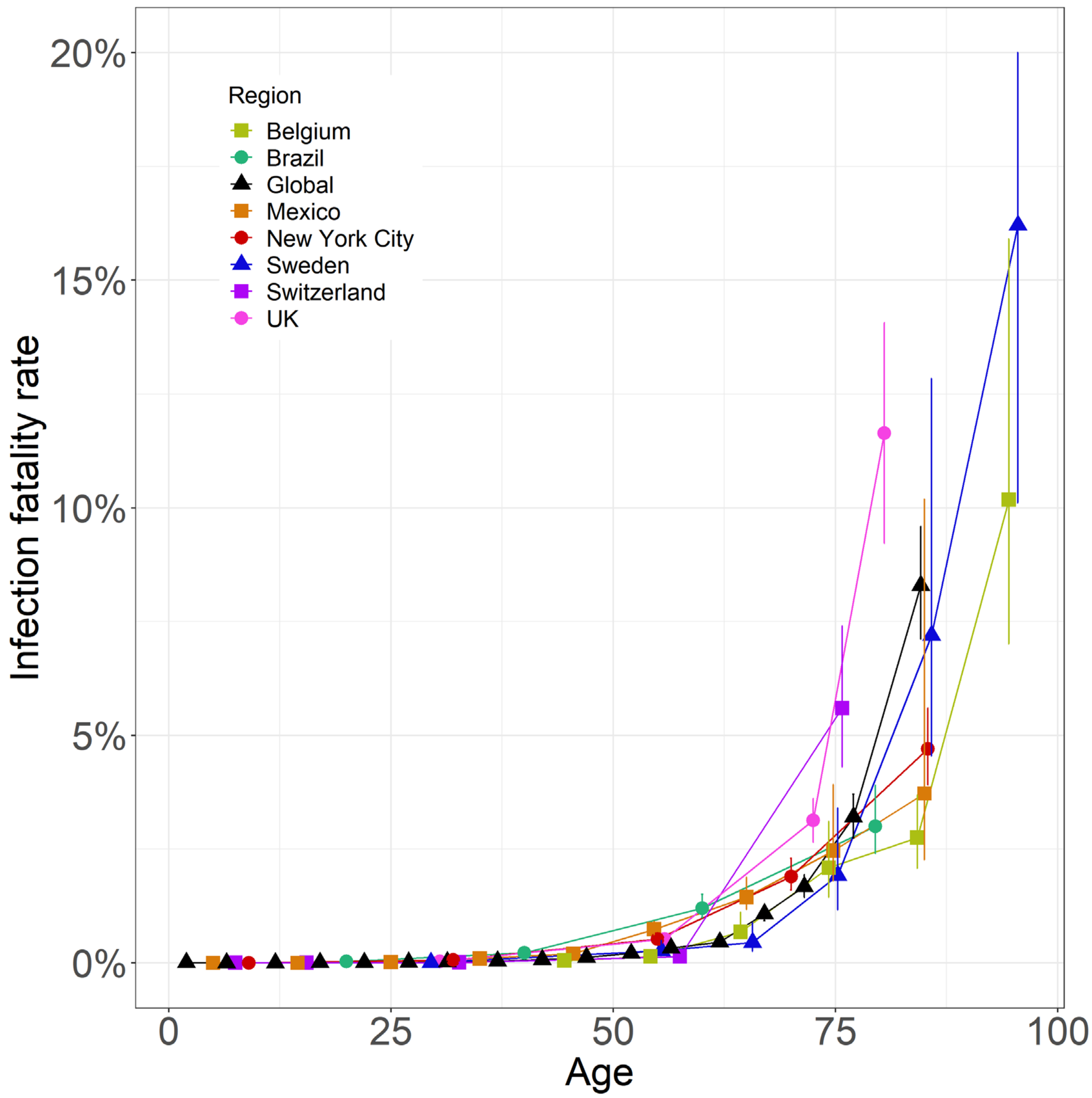

Supplement: S1 Fig — Points are plotted at the midpoint of the age class on the x-axis, and slightly jittered along the x-axis facilitate presentation. Lines show age-specific IFRs for different populations, using confirmed-only COVID-19 deaths where possible. This study is represented in red, as “New York City”. IFR means and confidence intervals that estimate a value of 0 are represented as 0.001. The upper bound on the 90+ class for Sweden extends to 29.5% but is represented here as 20%. (PDF) [file pone.0285612.s001.pdf]
