# Supplementary material for: Age-specific SARS-CoV-2 infection fatality rates derived from serological data vary with income and income inequality
Source: PLoS One. 2023 May 17;18(5):e0285612. doi: 10.1371/journal.pone.0285612 (PMC10191265; doi:10.1371/journal.pone.0285612)
Supplement: S2 Fig — Higher values of the Gini index represent higher levels of inequality. Fitted lines show significant relationships. Age classes include (a) 18–44, (b) 45–64, (c) 65–74, and (d) 75+. There were only three IFR estimates for the 0–17 age category so this age category was omitted from the plot. (PDF) [file pone.0285612.s002.pdf]

S2 Figure. Infection fatality ratio (IFR) of COVID-19 plotted against the Gini index [1]**.** Higher values of the Gini index represent higher levels of inequality. Fitted lines show significant relationships. Age classes include (a) 18-44, (b) 45-64, (c) 65-74, and (d) 75+. There were only three IFR estimates for the 0-17 age category so this age category was omitted from the plot.

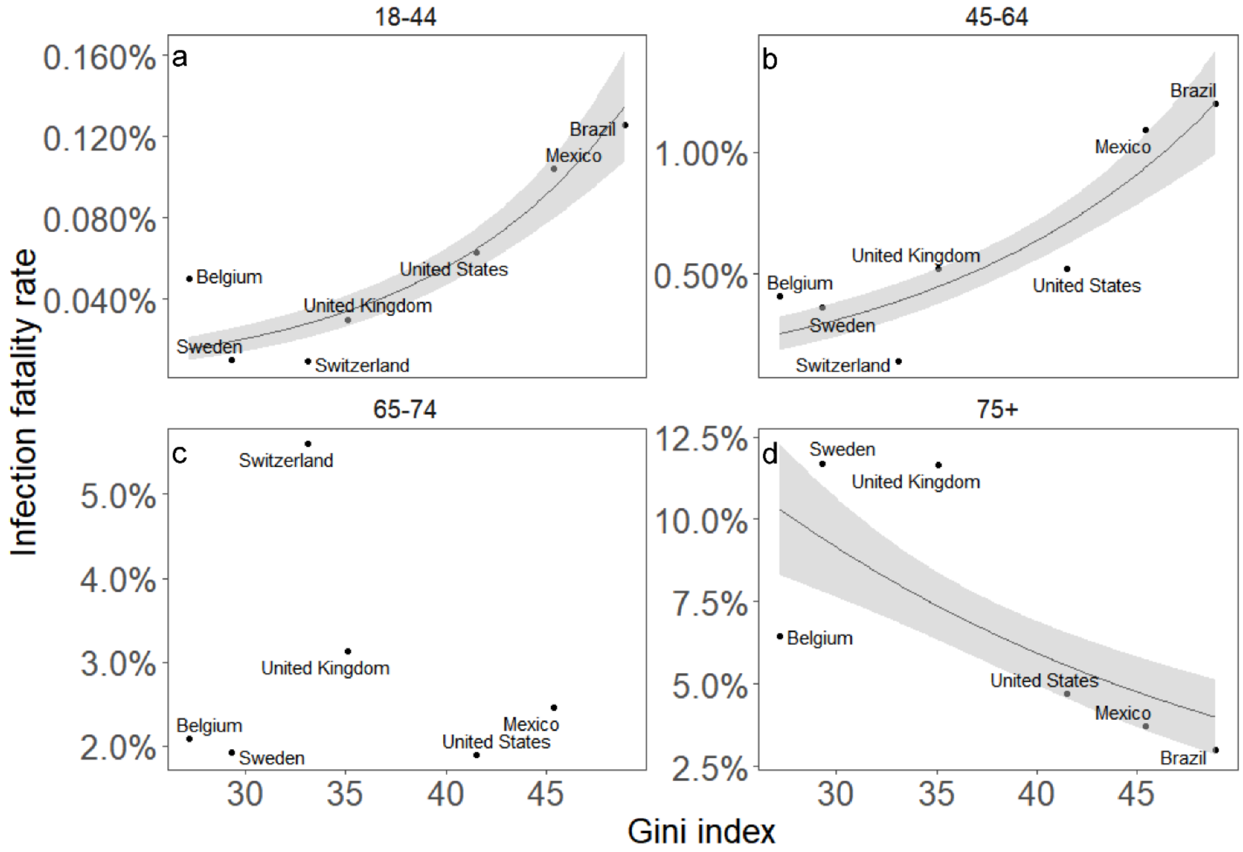

References

1. Gini index | Data [Internet]. [cited 2022 Jul 9]. Available from: https://data.worldbank.org/indicator/SI.POV.GINI
