# Supplementary material for: Age-specific SARS-CoV-2 infection fatality rates derived from serological data vary with income and income inequality
Source: PLoS One. 2023 May 17;18(5):e0285612. doi: 10.1371/journal.pone.0285612 (PMC10191265; doi:10.1371/journal.pone.0285612)
Supplement: S1 Table — The delays from symptom onset to case reporting [27] and from case reporting to death [28] are specific to New York City. See Perez-Saez et al. [11] for additional methods on delay distributions. (PDF) [file pone.0285612.s003.pdf]

S1 Table. Delay distributions and sources. The delays from symptom onset to case reporting [1] and from case reporting to death [2] are specific to New York City. See Perez-Saez et al. [3]for additional methods on delay distributions.

**Delay Description**	**Distribution**	**Source**
Infection to symptom onset (Incubation period)	Lognormal(μ = 1.57, σ = 0.65)	Bi et al. 2020 [4]
Symptom onset to case reporting	Lognormal(μ = 0.693, σ = 1)	Greene et al. 2021 [1]
Symptom onset to seroconversion	Lognormal(μ = 2.34, σ = 0.38)	Stringhini et al. 2020 [5]
Case reporting to death	Lognormal(μ = 2.08, σ = 1)	Thompson et al. 2020 [2]

References

1. Greene SK, McGough SF, Culp GM, Graf LE, Lipsitch M, Menzies NA, et al. Nowcasting for Real-Time COVID-19 Tracking in New York City: An Evaluation Using Reportable Disease Data From Early in the Pandemic. JMIR Public Health and Surveillance. 2021 Jan 15;7(1):e25538.

2. Thompson CN, Baumgartner J, Pichardo C, Toro B, Li L, Arciuolo R, et al. COVID-19 Outbreak — New York City, February 29–June 1, 2020. MMWR Morb Mortal Wkly Rep. 2020 Nov 20;69(46):1725–9.

3. Perez-Saez J, Lauer SA, Kaiser L, Regard S, Delaporte E, Guessous I, et al. Serology-informed estimates of SARS-CoV-2 infection fatality risk in Geneva, Switzerland. The Lancet Infectious Diseases. 2021 Apr 1;21(4):e69–70.

4. Bi Q, Wu Y, Mei S, Ye C, Zou X, Zhang Z, et al. Epidemiology and transmission of COVID-19 in 391 cases and 1286 of their close contacts in Shenzhen, China: a retrospective cohort study. Lancet Infect Dis. 2020 Aug;20(8):911–9.

5. Stringhini S, Wisniak A, Piumatti G, Azman AS, Lauer SA, Baysson H, et al. Seroprevalence of anti-SARS-CoV-2 IgG antibodies in Geneva, Switzerland (SEROCoV-POP): a population-based study. International Journal of STD and AIDS. 2020 Aug 1;396(10247):313–9.
