# Supplementary material for: Age-specific SARS-CoV-2 infection fatality rates derived from serological data vary with income and income inequality
Source: PLoS One. 2023 May 17;18(5):e0285612. doi: 10.1371/journal.pone.0285612 (PMC10191265; doi:10.1371/journal.pone.0285612)
Supplement: S2 Table — Serosurveys based on blood donors were not considered. (PDF) [file pone.0285612.s004.pdf]

S2 Table. Exclusion criteria for global age-specific IFR comparisons. Serosurveys based on blood donors were not considered.

Country	Author	Reason for exclusion
Portugal	Kislaya et al. [1]	Participants were recruited from hospital and laboratory network and likely biased towards people coming into those locations
Italy	Poletti et al. [2]	Low sample size of deaths (n < 10 for most age groups)
Germany, Canada, Spain	Wagner et al. [3], Tang et al. [4], Pastor-Barriuso et al. [5]	Did not account for delay between infection and death
India	Cai et al. [6]	Inconsistent mortality data
Kenya, Denmark	Uyoga et al. [7], Erikstrup et al. [8]	Based on blood donations
Global	Levin et al. [9], COVID-19 Forecasting Team [10]	Used a point estimate for delays between infection and death or seroconversion
New York State	Brazeau et al. [11]	IFR estimates were estimated as a part of a larger global analysis; no independent age-specific IFRs were estimated using local data and age-classes.

References

1. Kislaya I, Gonçalves P, Barreto M, Sousa R de, Garcia AC, Matos R, et al. Seroprevalence of SARS-CoV-2 Infection in Portugal in May-July 2020: Results of the First National Serological Survey (ISNCOVID-19). Acta Médica Portuguesa. 2021 Feb 1;34(2):87–94.

2. Poletti P, Tirani M, Cereda D, Trentini F, Guzzetta G, Marziano V, et al. Age-specific SARS-CoV-2 infection fatality ratio and associated risk factors, Italy, February to April 2020. Euro Surveill. 2020 Aug 6;25(31):2001383.

3. Wagner R, Peterhoff D, Beileke S, Günther F, Berr M, Einhauser S, et al. Estimates and Determinants of SARS-Cov-2 Seroprevalence and Infection Fatality Ratio Using Latent Class Analysis: The Population-Based Tirschenreuth Study in the Hardest-Hit German County in Spring 2020. Viruses. 2021 Jun;13(6):1118.

4. Tang X, Sharma A, Pasic M, Colwill K, Birnboim C, Nagelkerke N, et al. COVID Symptoms, Seroprevalence, and Mortality During the First Wave of SARS-CoV-2 in Canada [Internet]. Rochester, NY: Social Science Research Network; 2021 Jan [cited

2022 Apr 5]. Report No.: 3752659. Available from: https://papers.ssrn.com/abstract=3752659

5. Pastor-Barriuso R, Pérez-Gómez B, Hernán MA, Pérez-Olmeda M, Yotti R, Oteo-Iglesias J, et al. Infection fatality risk for SARS-CoV-2 in community dwelling population of Spain: nationwide seroepidemiological study. BMJ. 2020 Nov 27;371:m4509.

6. Cai R, Novosad P, Tandel V, Asher S, Malani A. Representative estimates of COVID-19 infection fatality rates from four locations in India: cross-sectional study. BMJ Open. 2021 Oct 1;11(10):e050920.

7. Uyoga S, Adetifa IMO, Karanja HK, Nyagwange J, Tuju J, Wanjiku P, et al. Seroprevalence of anti-SARS-CoV-2 IgG antibodies in Kenyan blood donors [Internet]. medRxiv; 2020 [cited 2022 Apr 5]. p. 2020.07.27.20162693. Available from: https://www.medrxiv.org/content/10.1101/2020.07.27.20162693v1

8. Erikstrup C, Hother CE, Pedersen OBV, Mølbak K, Skov RL, Holm DK, et al. Estimation of SARS-CoV-2 Infection Fatality Rate by Real-time Antibody Screening of Blood Donors. Clin Infect Dis. 2021 Jan 27;72(2):249–53.

9. Levin AT, Hanage WP, Owusu-Boaitey N, Cochran KB, Walsh SP, Meyerowitz-Katz G. Assessing the age specificity of infection fatality rates for COVID-19: systematic review, meta-analysis, and public policy implications. Eur J Epidemiol. 2020 Dec 1;35(12):1123–38.

10. COVID Forecasting Team. Variation in the COVID-19 infection–fatality ratio by age, time, and geography during the pre-vaccine era: a systematic analysis. The Lancet. 2022 Feb;S0140673621028671.

11. Brazeau N, Verity R, Jenks S, Fu H, Whittaker C, Winskill P, et al. Report 34: COVID-19 infection fatality ratio: estimates from seroprevalence [Internet]. Imperial College London; 2020 Oct [cited 2022 Mar 25]. Available from: http://spiral.imperial.ac.uk/handle/10044/1/83545
