# Supplementary material for: Age-specific SARS-CoV-2 infection fatality rates derived from serological data vary with income and income inequality
Source: PLoS One. 2023 May 17;18(5):e0285612. doi: 10.1371/journal.pone.0285612 (PMC10191265; doi:10.1371/journal.pone.0285612)
Supplement: S3 Table — The last column notes the percent increase in the IFR comparing confirmed deaths to confirmed and probable deaths. (PDF) [file pone.0285612.s005.pdf]

S3 Table. Seroprevalence and infection fatality rate (IFR) estimates in New York City for five age classes, using confirmed and probable COVID-19 deaths. The last column notes the percent increase in the IFR comparing confirmed deaths to confirmed and probable deaths.

Age class	Confirmed COVID-19 Deaths, as of May 17, 2020 [1]	IFR (95% CI), Confirmed Deaths	Combined (Confirmed + Probable) COVID-19 Deaths, as of May 17, 2020 [1]	IFR (95% CI), Combined Deaths	% increase from IFR estimates using confirmed deaths only
0-17	10	0.0015 (0.00038-0.0037)	13	0.0027 (0.00086 – 0.006)	80.0%
18-44	625	0.063 (0.052-0.076)	750	0.077 (0.062 – 0.094)	22.2%
45-64	3556	0.52 (0.43-0.61)	4490	0.67 (0.55 – 0.78)	28.8%
65-74	3963	1.9 (1.6-2.3)	4893	2.5 (2.1 – 3.0)	31.6%
75+	7731	4.7 (3.9-5.6)	10105	6.4 (5.3 – 7.6)	36.2%

References

1. COVID-19: Data Archive - NYC Health [Internet]. [cited 2022 Feb 28]. Available from: https://www1.nyc.gov/site/doh/covid/covid-19-data-archive.page
