# Supplementary material for: Age-specific SARS-CoV-2 infection fatality rates derived from serological data vary with income and income inequality
Source: PLoS One. 2023 May 17;18(5):e0285612. doi: 10.1371/journal.pone.0285612 (PMC10191265; doi:10.1371/journal.pone.0285612)
Supplement: Attachment — Submitted filename: Response to Reviewers.docx [file pone.0285612.s006.docx]

February 26, 2023

Dr. Junyuan Yang

Academic Editor

PLOS ONE

Dear Dr. Junyuan Yang,

We would like to thank you for your letter on February 20, 2023 and the opportunity to revise and resubmit our manuscript: “**Age-specific SARS-CoV-2 infection fatality rates derived from serological data vary with income and income inequality**” for publication as a Research Article in *PLOS ONE*.

In this manuscript, we show that infection fatality rates of COVID-19, estimated from previously published serological data, differ among developed countries even after controlling for age. This work will be of interest to epidemiologists and public health personnel, as well as the general audience of *PLOS ONE*.

Regarding the points raised by the editor:

1. The revised manuscript is formatted in accordance with PLOS ONE’s style requirements.
2. In this manuscript, we used published data which was collected under an IRB. This manuscript does not contain any original human research data. We have updated our Methods and Ethics Statement to clarify this.

Thank you for the consideration of this manuscript.

Sincerely,

Chloe Rickards

Department of Ecology and Evolutionary Biology

University of California, Santa Cruz

[rickards.chloe@gmail.com](mailto:rickards.chloe@gmail.com)

Dr. Marm Kilpatrick

Department of Ecology and Evolutionary Biology

University of California, Santa Cruz

[akilpatr@ucsc.edu](mailto:akilpatr@ucsc.edu)
